# Strategic use of dual regimens of boosted protease inhibitors plus maraviroc in poorly adherent subjects in view of long-acting drugs: A retrospective study

**DOI:** 10.1097/MD.0000000000005728

**Published:** 2017-02-17

**Authors:** Amedeo Ferdinando Capetti, Mariangela Micale, Laura Carenzi, Fosca Niero, Simona Landonio, Stefania Vimercati, Gianfranco Dedivitiis, Giuliano Rizzardini

**Affiliations:** a 1^st^ Division of Infectious Diseases; b Pharmacy Unit, Luigi Sacco Hospital, Milano, Italy; c Whitwaterstrand University, Johannesburg, South Africa.

**Keywords:** adherence, atazanavir, darunavir, maraviroc, resistance, strategic

## Abstract

In view of the forthcoming long-acting antiretrovirals, measures should be taken to prevent the selection of HIV drug resistance mutations. All subjects who had been switched to boosted protease inhibitors plus maraviroc (bPIs/MVC) with baseline HIV-1 RNA >50 copies/mL between June, 2014, and April, 2015, were retrospectively evaluated. HIV-1 RNA, CD4+ T-cells, serum glucose, creatinine, ALT, and adverse events were controlled every 3 to 4 months. We retrospectively analyzed 44 patients: 18 were taking darunavir/ritonavir (DRV/r) and 26 atazanavir/ritonavir (ATV/r) once daily, plus MVC 300 mg once daily. Seven subjects were in CDC stage C. All had a follow-up of at least 24 weeks, 28 exceeded 48 weeks, and 21 exceeded 72 weeks. All had experienced at least 1 viral failure and had selected at least 1 resistance-associated mutation (RAM). At baseline, 38 had plasma HIV-1 RNA 50-499 copies/mL and 6 had ≥500. At week 24, none had viremia >500 and 30 (68.2%) had suppressed HIV-1 RNA below 50 copies/mL. Of the subgroup with 48 weeks’ follow-up, 23 had HIV-1 RNA 50-499 copies/mL, 5 had ≥500, and 20/28 suppressed to <50 copies/mL. Of the longest observed subgroup (72 weeks), 17 had HIV-1 RNA 50-499 copies/mL, and 4 had ≥500 copies/mL and 15/21 (71.4%) suppressed to <50 copies/mL. This combination allowed fair suppression of viral replication, with minor genotypic evolution in 6 subjects, and seems to be a feasible strategy to prevent damaging future options.

## 1 Introduction

In most observational cohorts, there are patients who do not respond to measures to improve adherence to antiretroviral therapy.^[1]^ Their viremia often rebounds, accumulating resistance mutations, and multidrug resistance has been related to disease progression and death.^[2]^

In view of future strategies of long-acting antiretrovirals, composed of non-nucleoside reverse transcriptase inhibitors (NNRTIs) and integrase strand transfer inhibitors (INSTIs), measures should be taken to prevent the selection of mutations that would affect HIV sensitivity to such drugs.

The high genetic barrier to resistance of protease inhibitors (PIs)^[3,4]^ makes physicians confident that in case they prove to be ineffective, the risk of selecting resistance-associated mutations is low. Moreover, while effective viral suppression on therapy limits the switch from R5-tropic to non-R5-tropic strains, uncontrolled viral replication and immunologic decline lead to more frequent detection of dual/mixed tropism,^[2]^ a pattern where not only maraviroc (MVC) has no role to play, but also disease progression accelerates^[5–7]^ and CD4+ T-cell recovery is impaired.^[8]^ Therefore, MVC should be used early in these subjects, since this chance may be lost in later stages.

Studies on switch to bPI monotherapy in treatment-experienced subjects (mainly after durable viral suppression) showed inferior efficacy as compared to the continuation of combination antiretroviral therapy (cART).^[9,10]^ Trials on bPIs plus MVC dual regimens have yielded controversial results, both in naive subjects and in switch. The 96-week data from study A4001078 showed noninferiority of the dual combination of MVC plus ATV/r in naive subjects as compared to 2 nucleosides plus ATV/r, the only discontinuations being due to hyperbilirubinemia.^[11]^ After 24 weeks’ encouraging results,^[12]^ the GUSTA Study, comparing the continuation of a triple therapy with the switch to once daily MVC 300 mg plus DRV/r 800/100 mg, was interrupted due to excess treatment failures in the simplification arm.^[13]^ A retrospective analysis by Macías et al,^[14]^ however, showed that the switch to DRV/r 800/100 mg plus MVC 150 mg maintained the proportion of subjects suppressing HIV replication (86% vs 80% at baseline) after 48 weeks of therapy. Moreover, MVC in association with bPIs behaved fairly well in a wide observational analysis of simplification to dual therapies based on bPIs, being the second best companion drug after raltegravir.^[15]^ The present study is aimed to describe the initial results and durability of this regimen in preserving future options and suppressing viral replication as low as possible in a cohort of chronic nonsuppressors.

## 2 Population and methods

### 2.1 Patients’ population

To be considered poorly adherent and poorly responsive to adherence correction measures, the patients enrolled had to meet at least one of the following criteria, besides active replication (>50 copies/mL) for more than 6 months: having undergone relative-controlled directly observed therapy (DOT), or medically-assisted DOT, and/or having been admitted in ward to better control the response to therapy, and/or having been followed by a psychiatrist or a psychologist with no or transient improvement in virologic response.

All subjects meeting inclusion criteria and switched to bPIs/MVC with baseline HIV-1 RNA >50 copies/mL, followed at Luigi Sacco Hospital, 1st Division of Infectious Diseases outpatients’ clinic, Milan, Italy, between June, 2014, and April, 2015, were retrospectively evaluated. The local Ethics’ Committee was informed by letter as required by the Italian legislation on nonsponsored retrospective studies.

### 2.2 Intervention

After the detection of R5-tropic virus at a genotypic tropism test, interpreted according to the geno2pheno algorithm, version 3.4,^[16]^ patients signed an informed consent with privacy disclosure approval, as this is a nonconventional (although not off-label) antiretroviral regimen, and were switched to a boosted PI (either atazanavir 300 mg or darunavir 800 mg plus ritonavir 100 mg once daily) plus maraviroc 300 mg once daily, except 2 older patients who took maraviroc 150 mg once daily having eGFR <80 mL/min.

### 2.3 Endpoints

HIV-1 RNA (Abbott HIV-1 RT PCR Kit), CD4+ T-cells, serum glucose, serum creatinine and ALT and adverse events were monitored at least every 3 to 4 months as per routine clinical follow-up. Patients were instructed to present on a monthly basis for virologic and clinical check-up until optimal viral control was reached (<50 copies HIV-1 RNA/mL).

The main variable of the study was the patients’ adherence, which was only self-reported in terms of number of doses missed in the last month. Since this population is particularly risky and difficult to manage, it was strictly controlled by physicians, so that no one missed the main timelines for control (± 2 weeks).

### 2.4 Statistical analysis

The population size was determined by the number of patients corresponding to the definition in our outpatients’ clinic, excluding those (n = 16) who had planned a >2 months’ stay in their countries of origin (mainly Hispanics returning to South America), and those (n = 12) who had missing genotypic tests at previous failure for any reason. Selected patients came out to be all Caucasian and 43 harbor HIV-1 subtype B, whereas one has subtype F.

Apart from CD4 mean ± standard deviation (SD), the only statistical analysis performed was the 2-tailed Fisher exact test, for reaching <50 copies/mL between the ATV/r and the DRV/r group, given that the small population was powered to detect only major differences.

When reporting virologic and immunologic results, the subpopulations who reached at least 48 and 72 weeks of follow-up were treated as separate groups, so that data at each time-point reflects the single population studied rather than the progressive narrowing of a large baseline.

## 3 Results

We analyzed 44 patients with at least 24 weeks of follow-up: 18 were taking once daily DRV/r and 26 were taking ATV/r in association with 300 mg of MVC once daily. The identification of double/mixed tropic HIV-1 prevented the inclusion of 2 additional subjects on ATV/r-based regimens and 3 on DRV/r-based regimens. Males were 77.3%, n = 34, non-Caucasians 9.1%, n = 4, all hispanic, and the mean age was 48.2 years. CDC stage C had been diagnosed to 7 (15.9%) subjects. Minor HIV-1-related symptoms, such as oral hairy leukoplakia, seborrheic dermatitis, weight loss, fever, and fatigue were present at baseline in 6 patients (13.6%). The main risk factors were quite balanced (males having sex with males 36.4%, n = 16, past or ongoing drug addiction 34.1%, n = 15, and heterosexual intercourse 29.5%, n = 13). Table 1 shows the demographic and baseline parameters of the 2 cohorts of protease inhibitors.

**Table 1:**
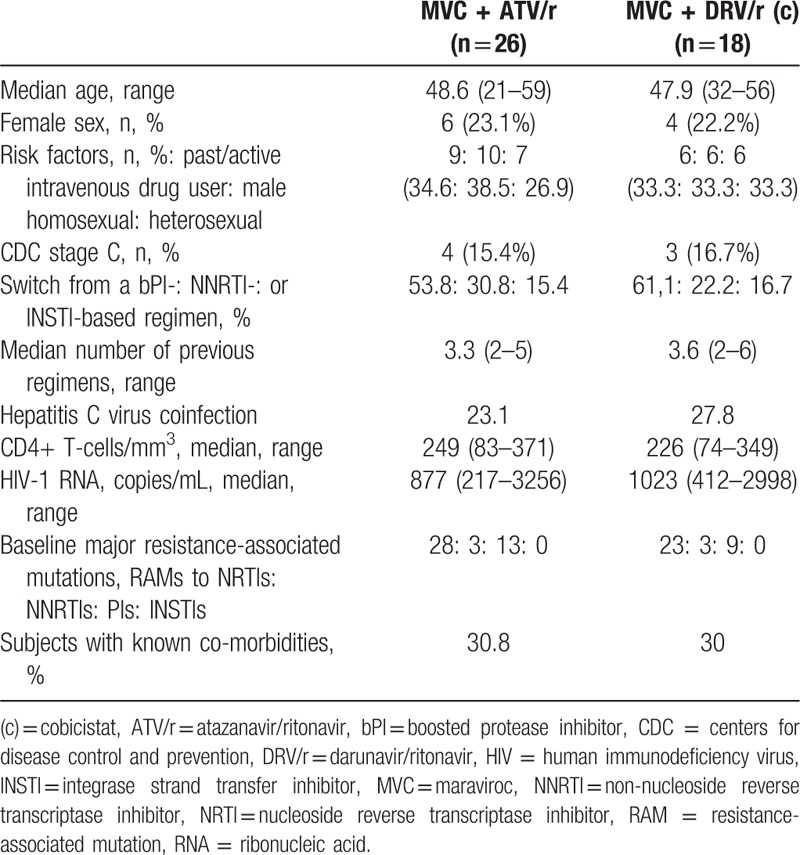
Baseline characteristics of the population, divided for the 2 bPIs.

**Figure 1:**
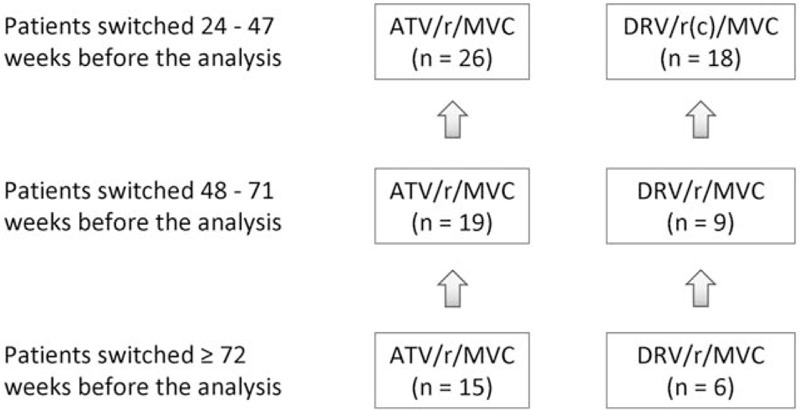
Flow diagram of the population divided by the protease inhibitor.

All subjects had a follow-up of at least 24 weeks, 28 exceeded 48 weeks, and 21 exceeded 72 weeks. None has discontinued the dual regimen up to date and none has been lost to follow-up (see Fig. 1). Only 5 subjects taking darunavir switched in the last 2 months from booster ritonavir to cobicistat.

**Figure 2:**
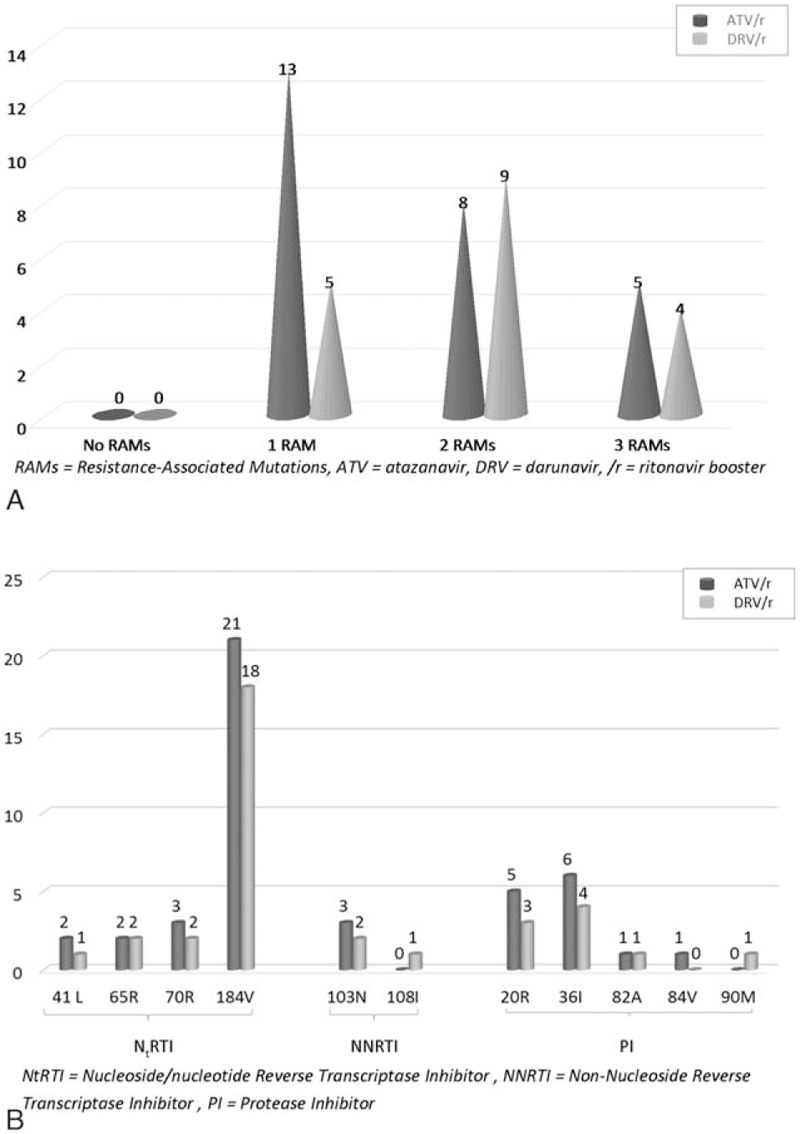
Baseline RAMs at the time of switch: (A) n. patients per number of mutations; (B) n. patients per each mutation, divided for the main classes of antiretrovirals. RAM = resistance-associated mutation.

All had at least 1 failure in their treatment history, and 43.2% (n = 19) had several failures. All had at least 1 resistance-associated mutation (RAM), mostly to NRTIs, as described in Fig. 2. Six subjects were taking proton pump inhibitors and the suggested timing was 3 to 4 hours after antiretroviral therapy dosing.

**Figure 3:**
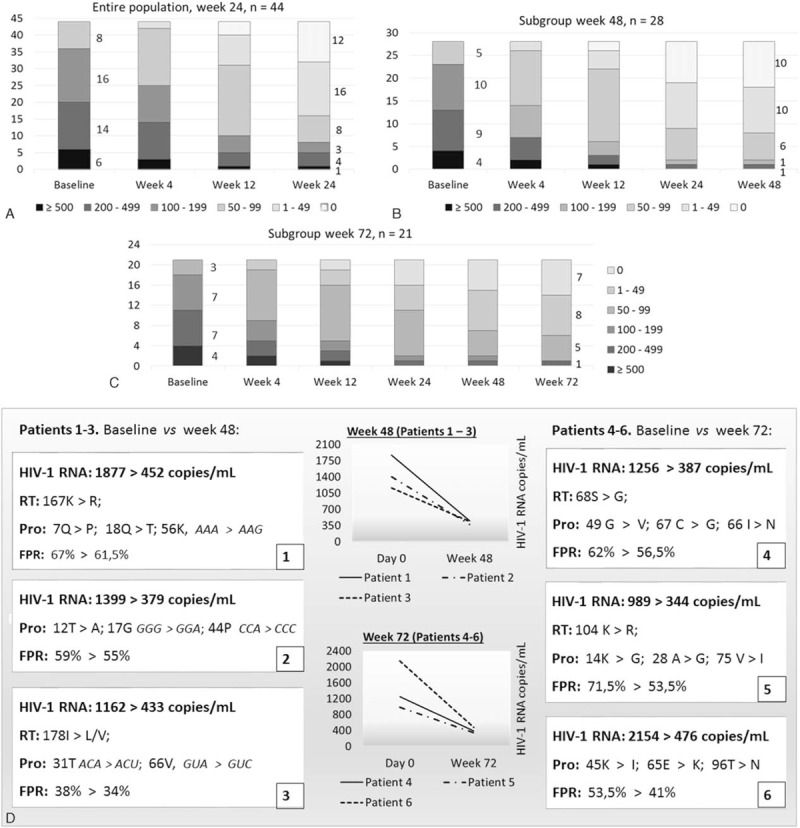
(A–C) HIV-1 RNA decay over time by ranges of values; (D) evolution of HIV-1 sequence mutations (synonimous codons reported) and tropism in patients responding poorly to the strategy.

At baseline 38 had plasma HIV-1 RNA 50–499 copies/mL and 6 had ≥500. At week 24 none had viremia >500 and 30 had suppressed below 50 copies/mL. Of the subgroup with 48 weeks’ follow-up, 8 had HIV-1 RNA 50–499 copies/mL and 20/28 suppressed to <50 copies/mL. Of the longest observed subgroup (72 weeks), 5 had HIV-1 RNA 50–99 copies/mL and one 200–499 copies/mL and 15/21 suppressed to <50 copies/mL. The odds ratio of reaching <50 copies HIV-1 RNA/mL at week 24 between the ATV/r and DRV/r groups was 0.89 and the 2-tailed Fisher exact test was not significant (*P* = 1). The detailed virologic evolution of the cohort is described in Fig. 3.

Patients who did not reach <50 copies HIV-1 RNA had poorer adherence than those who did. In the former population, the median number of doses missed in a month was 7 (range 4 – 9), compared to 2 in the latter group (range 1–3). The overall median number of doses missed in the last month was 4 (range 1 – 9), whereas only the 12 subjects whose HIV-1 RNA steadily declined to “nondetected” claimed to be fully adherent. Tropism false positive rates (FPR) in 2 subjects who maintained active viral replication declined by about 5% at week 48 and in other 2 by about 15% at week 72; however, the range was still 41% to 61.5%, well above the 10% suggested by the European guidelines for discriminating dual/mixed/X4-tropic strains. Few synonymous and nonsynonymous polymorphic mutations appeared in 6 subjects who maintained HIV-1 RNA >300 copies/mL, indicating active replication.

**Figure 4:**
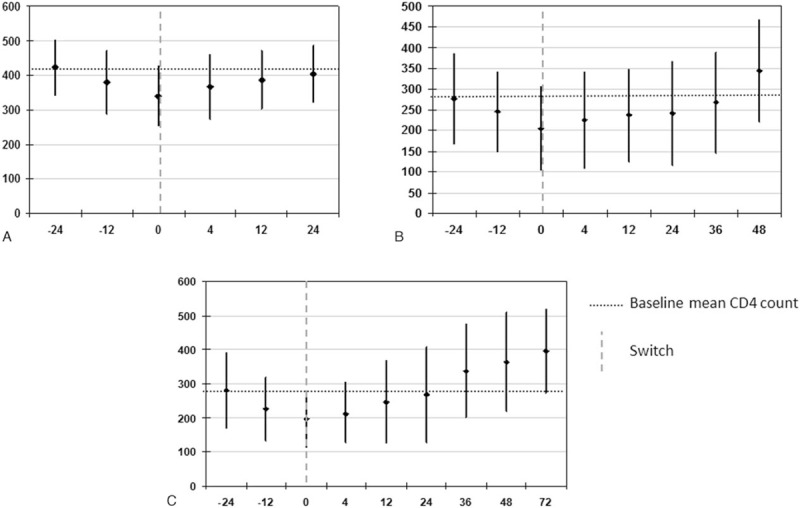
CD4+ T-cell absolute recovery after decay in (A) the entire population (24 weeks, n = 44), (B) the 48-weeks subgroup (n = 28), and (C) the 72-weeks subgroup (n = 21), mean ± SD values. SD = standard deviation.

The CD4+ T-cell recovery, following the 24 weeks’ decay observed before the strategy was introduced, is outlined in Fig. 4.

## 4 Discussion

The use of MVC and a bPI reduced the proportion of subjects with uncontrolled HIV-1 replication and prevented the emergence of major HIV drug resistance mutations over the observation period.

Evident limitations are the small patient population and the absence of a randomized control arm; however, compared to the patients’ previous 24 weeks, CD4+ T- cells increased and the salvage treatment options did not narrow. It is possible that the dramatic yet realistic presentation of this switch by the treating physicians, who emphasized the serious shortage of treatment options and the possible impact on survival, and the need of a gating test may have temporarily increased the patients’ motivation towards adherence and we cannot distinguish this from the effect of the regimen itself.

Moreover, this treatment regimen theoretically has a series of metabolic advantages that may determine a reduction of the risk of long-term metabolic toxicity. MVC reduces ritonavir-induced atherogenesis in animal studies,^[17]^ an effect shown also by ATV/r on HIV-infected subjects^[18]^ and several analyses have suggested MVC potential to downregulate inflammation, particularly at high concentrations.^[19]^ Therefore, given its’ wide therapeutic window and excellent tolerability profile, we decided to utilize MVC 300 mg instead of the 150 mg suggested in association with bPIs. ATV/r has shown to have a better impact on carotids’ intima-media thickness as compared to DRV/r and raltegravir at 144 weeks in the ACTG substudy A5260 s.^[20]^ Finally, from a pharmaco-economic point of view, the cost of MVC at these doses is comparable to that of a fixed dose combination of nucleoside/nucleotide analogs.

## 5 Conclusions

The association of bPIs plus MVC allowed fair suppression of viral replication (68.2% BLD by week 24, rising to 71.4% in both longer observation subgroups), with only minor genotypic evolution in 6 subjects, confirming that it is a feasible strategy for delaying the risk of damaging future options in view of long-term regimens, ideal for such patients.
